# Acute pericarditis after ovarian stimulation in a patient with systemic lupus erythematosus: a case report and literature review

**DOI:** 10.3389/fimmu.2025.1715055

**Published:** 2025-11-26

**Authors:** Haihong Wang, Li Deng, Changjuan Shen, Zhenhua Wen, Yu Zou, Yang Cao

**Affiliations:** 1 Department of Pharmacy, School of Medicine, Institute of Pharmaceutical Process, Wuhan University of Science and Technology, Wuhan, Hubei, China; 2 Department of Rheumatology and Immunology, Zhuzhou Hospital Affiliated to Xiangya Medical College, Central South University, Zhuzhou, Hunan, China; 3 School of Health Humanities, Hubei University of Chinese Medicine, Wuhan, Hubei, China; 4 Hubei Shizhen Laboratory, Wuhan, Hubei, China

**Keywords:** acute pericarditis, ovarian stimulation, systemic lupus erythematosus (SLE), ovulation induction (OI), controlled ovarian stimulation (COS)

## Abstract

Acute pericarditis is a clinical syndrome characterized by acute inflammation of the pericardium, which may result from various infectious or non-infectious etiologies. The characteristic clinical manifestations primarily encompass chest pain, classic electrocardiographic changes, and new or worsening pericardial effusion. Acute pericarditis may manifest independently or as a component of systemic disease. Systemic lupus erythematosus (SLE)-associated pericarditis typically occurs during active disease phase. Ovarian stimulation promotes ovulation and alters sex hormone levels, potentially triggering or exacerbating SLE. We report a 34-year-old woman with stable SLE who developed a disease flare, predominantly manifesting as acute pericarditis, following ovarian stimulation. Significant clinical improvement was observed following treatment with glucocorticoids, colchicine, and immunosuppressive agents. A literature review identified eight additional cases of SLE flares following ovarian stimulation, presenting with arthritis, rash, pericarditis, nephritis, or thrombotic events. Consequently, a comprehensive recurrence risk assessment and close monitoring of disease activity are recommended for SLE patients undergoing this fertility treatment.

## Introduction

Ovarian stimulation refers to a medical intervention aimed at regulating ovarian function to promote follicle development and ovulation. Depending on the therapeutic objective and the expected number of follicles, it can be divided into two main strategies: ovulation induction (OI) and controlled ovarian stimulation (COS). OI is primarily indicated for patients with ovulatory disorders and uses oral or injectable medications to mimic the natural ovulatory process. This approach stimulates the maturation and subsequent ovulation of a single or limited number of follicles, with the goal of increasing the likelihood of conception. COS is a critical component of assisted reproductive technology (ART) and primarily involves the administration of exogenous gonadotropins to promote the development of multiple ovarian follicles. To prevent premature ovulation triggered by an endogenous luteinizing hormone (LH) surge, pituitary suppression agents are also used during COS to inhibit the natural ovulatory cycle (1, 2).

SLE is a chronic, systemic autoimmune disease that most frequently affects women of reproductive age between 15 and 44 years (3, 4). Although SLE per se does not impair fertility, compromised reproductive capacity in women with SLE may result from multiple factors, including advanced maternal age at conception, adverse effects of medications, environmental triggers, and major organ damage (5, 6). Various methods of ovarian stimulation commonly elevate the serum gonadotropin and estrogen levels. Elevated serum gonadotropin levels may act as a triggering factor for SLE flares. Simultaneously, high estrogen levels modulate the function of various immune cells via estrogen receptor (ERα and ERβ) signaling, resulting in immune dysregulation that induces or exacerbates SLE (7, 8).

Acute pericarditis is an acute inflammatory condition of the visceral and parietal layers of the pericardium. The characteristic clinical manifestations include sudden, sharp retrosternal or precordial pain, exacerbated by coughing, deep inspiration, or positional changes but relieved by sitting forward. Electrocardiogram (ECG) serves as a valuable diagnostic tool, typically demonstrating diffuse concave-upward ST-segment elevation, PR-segment depression, and a relatively specific downsloping TP-segment (Spodick’s sign). Echocardiographic detection of new or worsening pericardial effusion supports the diagnosis of pericarditis, but not all cases present with pericardial effusion (9–11). SLE patients are at high risk for concurrent pericarditis, which is significantly correlated with overall disease activity (12).

This case report describes a 34-year-old nulligravida woman with clinically stable SLE who developed a severe disease flare following ovarian stimulation. The flare manifested primarily as acute pericarditis, featuring positional chest pain, ECG changes (including ST-segment elevation, PR-segment depression, and a downsloping TP-segment), and new pericardial effusion. It was also accompanied by a marked elevation in C-reactive protein (CRP). In order to enhance clinicians’ recognition of SLE reactivation secondary to ovarian stimulation and to better guide fertility management in SLE patients, we present the patient’s detailed diagnostic and therapeutic course, complemented by insights from relevant literature.

## Case presentation

The patient was a 34-year-old nulligravida Chinese woman. She had a smoking history of over ten years, averaging ten cigarettes per day, and a body mass index (BMI) of 27.0 kg/m². She was admitted in early February 2025 with non-erosive polyarthritis involving bilateral knees, elbows, and wrists persisting for over one month, with associated morning stiffness lasting more than 60 minutes. Laboratory tests revealed the following: an antinuclear antibody (ANA) titer of 1:320 with a speckled pattern; a positive anti-Smith antibody; negative results for rheumatoid factor, anti-CCP antibody, anti-dsDNA, anticardiolipin (aCL), anti-β2-glycoprotein I antibodies (anti-β2GPI), and lupus anticoagulant (LA); an elevated CRP level of 12.4 mg/L (0-6.0); and an erythrocyte sedimentation rate (ESR) of 27 mm/hr (0–20). Urinalysis, complete blood count (CBC) and complement levels (C3 and C4) were within normal limits. The echocardiography was normal. These findings fulfilled the 2019 EULAR/ACR classification criteria, establishing the definitive diagnosis of SLE, with an SLE Disease Activity Index 2000 (SLEDAI-2K) score of 4. Following the initiation of therapy with prednisone 5 mg daily, tacrolimus 1 mg twice daily, and hydroxychloroquine 200 mg twice daily, a significant resolution of polyarthritis was observed, with subsequent maintenance of this regimen.

Due to fertility desires, the patient planned to pursue ART. During her menstrual phase in March 2025, the patient underwent ovarian reserve assessment at a local obstetrics and gynecology specialty hospital. Laboratory tests revealed an anti-Müllerian hormone (AMH) level of 1.64 ng/ml (0.18-11.17), a follicle-stimulating hormone (FSH) level of 6.08 mIU/ml, and an estradiol level of 50.9 pg/ml. Transvaginal ultrasound demonstrated an antral follicle count of 8–9 in the left ovary and 4 in the right ovary. From April 11 to 13, 2025, the patient underwent ovarian stimulation at another facility without disclosing her SLE diagnosis or modifying her immunosuppressive regimen. The ovarian stimulation protocol included urofollitropin 150 IU subcutaneously daily, menotropins 225 IU intramuscularly daily, somatropin 4 IU subcutaneously daily, and medroxyprogesterone 6 mg orally daily. Following the cessation of ovarian stimulation, a repeat serum estradiol level was 2211.51 pg/mL. Several days later, the patient experienced recurrence of systemic polyarthritis, predominantly affecting the bilateral knees, elbows, and wrists. The patient did not prioritize these symptoms and sought no medical attention. On April 22, 2025, she developed fever (peak temperature 39°C) accompanied by chest pain without an identifiable trigger. The pain was characterized as sharp, radiating from the mid to lower sternum to the left shoulder, and exacerbated by physical activity, which prompted an emergency department visit. Laboratory results revealed an elevated D-dimer level of 1.45 μg/mL (0-0.5). However, cardiac enzyme panels, ECG, and contrast-enhanced computed tomography (CT) of the chest revealed no abnormalities. The chest pain was partially alleviated following acetaminophen administration. By April 24, 2025, the patient continued to experience fever, and her chest pain had intensified. The pain was relieved in a sitting position and worsened when lying flat. Due to intolerable discomfort, she returned to the emergency department on the evening of April 25, 2025, and was subsequently admitted to the Department of Rheumatology and Immunology at our hospital.

**Figure 1:**
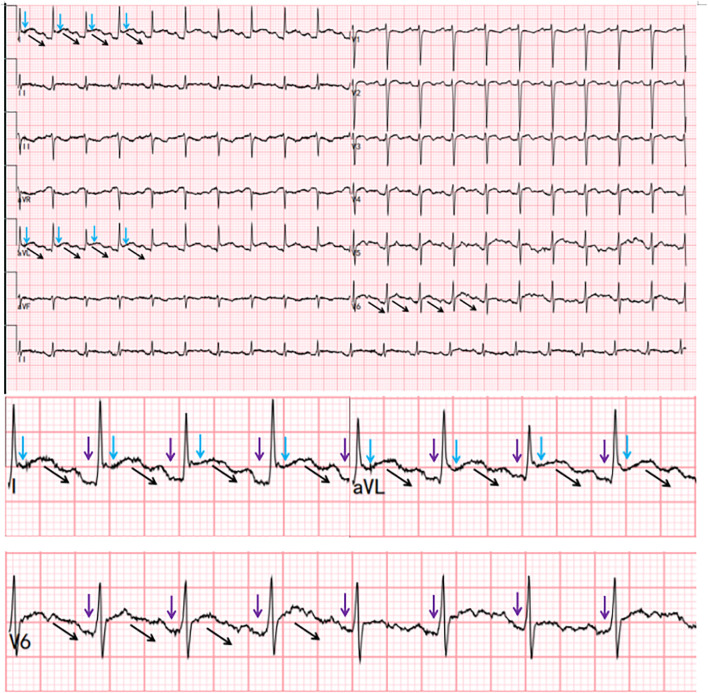
Concave-upward ST-segment elevation (blue arrow), PR-segment depression (purple arrow), and Spodick’s sign (black arrows).

**Figure 2:**
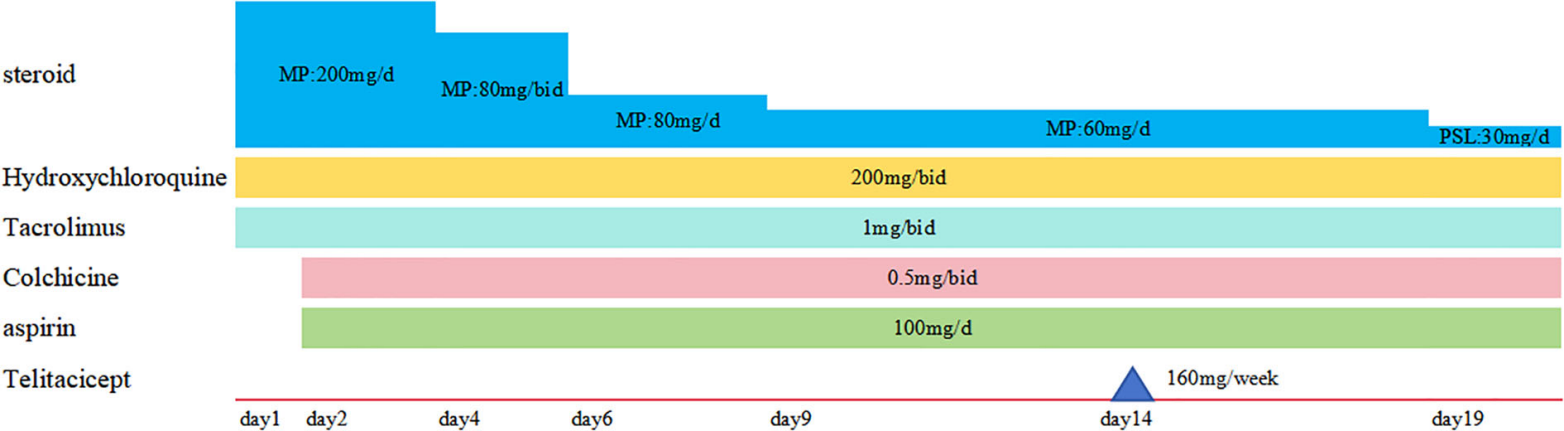
Therapeutic regimen (MP, Methylprednisolone; PSL, prednisolone).

After admission, an ECG revealed multilead concave-upward ST-segment elevation, PR depression, and Spodick’s sign (**Figure 1**). Acute ST-segment elevation myocardial infarction (STEMI) was suspected, but urgent coronary angiography showed no significant stenosis. Cardiac enzymes and troponin levels were normal. Bedside echocardiography revealed pericardial effusion (anterior pericardium 6 mm, posterior pericardium 11 mm, lateral pericardium 11 mm, right atrial roof 8 mm, cardiac apex 5 mm, right ventricular free wall 5 mm). Laboratory results were as follows: hemoglobin 98 g/L (115–150); white blood cell (WBC) count 18.91×10^9^/L (3.5-9.5) with neutrophil count 17.90×10^9^/L (1.8-6.3) and lymphocyte count 0.61×10^9^/L (1.1-3.2); platelet count 270×10^9^/L (125–350); CRP 313.3 mg/L; and ESR 117 mm/h. Hepatic and renal function tests as well as urinalysis were within normal limits. Serology was negative for anti-dsDNA, aCL, and anti-β2GPI, with normal complement C3 and C4 levels. LA was positive. Coagulation studies demonstrated a prothrombin time (PT) of 15.6 s (11.0-14.5) and an activated partial thromboplastin time (APTT) of 56.0 s (26.0-45.0). The patient presented with typical pericarditic chest pain, characteristic ECG changes, and new pericardial effusion, fulfilling the 2015 ESC pericarditis diagnostic criteria (13). The final diagnosis was acute pericarditis secondary to SLE, complicated by newly detected LA positivity, with an SLEDAI-2K score of 7. The patient was treated with intravenous methylprednisolone 200 mg once daily for 3 consecutive days, followed by a tapering regimen. Concurrent treatment included colchicine 0.5 mg twice daily and aspirin enteric-coated tablets 100 mg once daily. On April 28, 2025, the patient’s chest pain had alleviated compared with pretreatment levels, but remained significant when lying flat. Laboratory tests showed a significant decrease in inflammatory markers, with CRP reduced to 108.8 mg/L. A follow-up ECG on April 30 showed no abnormalities, and echocardiography indicated a marked reduction in pericardial effusion compared with previous findings (3 mm posterior and lateral to the left ventricle; 3 mm at the right atrial roof). On May 8, subcutaneous telitacicept 160 mg once weekly was added. The patient was discharged on May 13 with symptoms completely resolved. Before discharge, laboratory tests demonstrated: WBC count 9.81×10^9^/L; lymphocyte count 2.24×10^9^/L; CRP 6.6 mg/L; and ESR 32 mm/h. The treatment regimen is detailed in **Figure 2**.

One month post-discharge, the patient reported no recurrence of arthritis or chest pain. Laboratory investigations, including urinalysis, CBC, ESR, CRP, complement C3, and complement C4, were within normal limits. The patient achieved clinical quiescence, with an SLEDAI-2K score of 0. The prednisone dosage was then tapered to 20 mg/day. At the five-month follow-up, the patient’s condition was stable, and the prednisone dose was further tapered to 7.5 mg/day.

## Discussion

In this case, the patient developed multisystem involvement following ovarian stimulation, characterized by elevated estradiol levels, increased WBC count, fever, arthritis, and acute pericarditis. In SLE patients, it is essential to differentiate between a disease flare induced by ovarian stimulation and ovarian hyperstimulation syndrome (OHSS), as the latter may also manifest with multisystem involvement—including elevated estrogen levels, dyspnea, effusions in multiple serous cavities, and thrombotic events. However, OHSS does not exhibit the characteristic immunological abnormalities associated with active SLE, and its primary symptoms are gastrointestinal, such as abdominal distension. Serous cavity effusions predominantly manifest as ascites and pleural effusion, with isolated pericardial effusion being a rare occurrence. Ovarian enlargement is a distinctive imaging feature of OHSS (14). COS generally entails the administration of high-dose gonadotropins to induce concurrent development of multiple follicles. Various stimulation protocols can be formulated using different medication combinations. The choice of protocol is determined by individualized patient factors, including age, BMI, and ovarian reserve (2, 15). According to the European Society of Human Reproduction and Embryology (ESHRE) guidelines for ovarian stimulation in IVF/intracytoplasmic sperm injection (ICSI), while it remains uncertain whether gonadotropin doses exceeding 150 IU are recommended for predicted poor responders, the use of doses higher than 300 IU is not advised (16). High-dose stimulation may lead to multiple follicular development and a sharp rise in estrogen levels, thereby increasing the risk of OHSS and thromboembolic events. Based on the patient’s age, relevant risk factors, and her results for AMH and AFC, she did not meet the Bologna criteria for a predicted poor ovarian responder (17). Nevertheless, the patient was administered a COS regimen with a total daily gonadotropin dose of 375 IU. This protocol resulted in a marked elevation of her serum estradiol level to 2211.51 pg/mL, which precipitated a flare of SLE. When performing ovarian stimulation in SLE patients, clinicians must consider not only the success rate of ovarian stimulation but also maternal safety. A low-dose, mild stimulation protocol is preferred. Before initiating ovarian stimulation, a comprehensive treatment plan should be developed jointly with a rheumatologist, and prophylactic anticoagulation should be considered. New-onset SLE following ovarian stimulation has also been reported in healthy individuals, with similar pathogenic mechanisms.

Pericarditis is the most common cardiovascular manifestation of SLE, affecting approximately 20% of patients with SLE (18). Previous studies have reported that SLE patients with lymphocytopenia or positivity for antiphospholipid antibodies are at a higher risk of developing pericarditis, which is consistent with the presentation in this case (19). In most SLE patients, CRP levels typically remain normal even during active disease, and only mild elevation is observed in cases of SLE-associated arthritis. However, previous studies have indicated that SLE-related acute pericarditis may be accompanied by a marked increase in CRP. As demonstrated in the present case, the patient’s CRP reached a peak level of 313.3 mg/L (20, 21). Approximately 20%–30% of patients with acute pericarditis experience recurrence. Patients with elevated CRP levels or a history of recurrence are at a higher risk of disease relapse (22, 23).

Both acute pericarditis and STEMI are common causes of acute chest pain and ST-segment elevation on ECG. Given their significant differences in pathophysiology, management, and prognosis despite similar ECG presentations, accurate differential diagnosis is of paramount importance. Previous studies have indicated that approximately 19%–25% of patients with acute pericarditis may be misdiagnosed as STEMI (24). In acute pericarditis, ST-segment elevation is typically concave upward, diffuse, and involves most leads except aVR and V1. In contrast, STEMI usually demonstrates convex upward ST elevation, which is often localized to contiguous leads corresponding to the specific ischemic myocardial territory. In clinical practice, some early STEMI cases may present with concave upward ST-segment elevation, while a minority of pericarditis cases (especially focal pericarditis) might exhibit more regional and non-typical forms of ST-segment elevation, leading to diagnostic confusion (25–27). Spodick’s sign was considered a characteristic electrocardiographic change in pericarditis and was found in 80% of cases of pericarditis (28). But the latest report showed that the frequency of Spodick’s sign was 29% in pericarditis and 5% in STEMI (29). Compared with the diagnostic criteria in 2015, the 2025 diagnostic criteria for pericarditis consider clinical manifestations as a mandatory condition and elevate inflammatory markers, such as CRP, and cardiac magnetic resonance (CMR) findings to additional criteria (30). Although characteristic electrocardiographic changes support the diagnosis, they are not sufficient or necessary criteria. Given these diagnostic challenges, physicians should integrate dynamic ECG changes, troponin levels, echocardiography/CMR findings, and clinical signs for differential diagnosis. Coronary angiography may be performed when necessary.

NSAIDs and colchicine are generally first-line therapies for idiopathic or recurrent pericarditis. However, when pericarditis occurs as part of an SLE flare, glucocorticoids should be added to control systemic disease. According to the latest 2025 American College of Rheumatology (ACR) guidelines, pulse high-dose glucocorticoid therapy may be considered in SLE patients with organ-threatening or life-threatening situations (31, 32). Colchicine may be used in both acute and recurrent pericarditis and plays a vital role in reducing recurrence. Although a growing body of evidence supports the use of IL-1 blockers for the treatment of idiopathic or recurrent pericarditis, clinical data regarding their application in SLE-associated acute pericarditis remains insufficient (22, 33). Our patient also achieved satisfactory therapeutic effects using a B-cell targeted biologic agent unique to China (Telitacicept); however, future validation with a larger sample size is still needed.

Through systematic literature review, clinical data from eight well-documented cases were extracted and synthesized in **Table 1**. All patients maintained stable disease status before initiating ovarian stimulation therapy. Among them, five patients (Patients 1, 2, 3, 5, and 6) experienced mild flares of SLE, primarily manifested as arthritis, rash, and alopecia. Two patients (Patients 5 and 7) tested positive for antiphospholipid antibodies, with Patient 7 exhibiting severe complications such as inferior vena cava and left renal vein thrombosis. Patient 4 had two severe SLE flares, and Patient 8, with comorbid antiphospholipid syndrome (APS), developed lupus nephritis.

**Table 1:** Case reports of SLE flares after ovarian stimulation.

Patient	Age	Prior diagnosis	Primary disease treatment	Protocol & cycles	Complications	Reference
1	unknown	SLE	No therapy	Clomiphene & 2 Cycles	Discoid rash, ulcer	(34)
2	unknown	SLE	No therapy	Clomiphene, hMG & 6 Cycles	Arthritis	(34)
3	unknown	SLE	Prednisone	Unknown & 3 Cycles	Rash, myositis, vasculitis, alopecia	(34)
4	35	SLE	No therapy	Leuprorelin, hMG & 5/11 Cycles	1st flare: Pericarditis 2st flare: nephritis and arthritis	(35, 36)
5	31	SLE, aCL antibodies and LA positive	Prednisone, hydroxychloroquine, Aspirin	Leuprorelin, hMG &1 Cycle	Arthritis	(35, 36)
6	33	SLE	No therapy	Triptorelin, FSH, hMG & 3 Cycles	Discoid lesions and arthritis	(36)
7	27	SLE, aCL antibodies positive	Prednisone	hMG & 1 Cycle	Inferior vena cava and left renal vein thrombosis	(35, 36)
8	34	SLE, APS	Prednisone, hydroxychloroquine, aspirin	Unknown	Nephritis	(37)

Reproductive health remains a significant concern for patients with SLE, who face unique challenges related to disease activity and treatment. Simultaneously, advances in ovulation induction and ART have opened new avenues for SLE patients with impaired fertility. However, current evidence is largely based on uncontrolled studies, which, while suggesting the safety of these procedures, underscores the need for more robust data (38, 39). This experience of the patient in our case illustrates that even mild SLE in a stable condition can worsen after ovarian stimulation, with rapid and substantial changes in estrogen levels caused by ovulation-stimulating agents being identified as the primary underlying cause. Furthermore, elevated estrogen levels may predispose individuals to thrombotic events, as illustrated by Patient 7 in the literature, whereas in our case, the patient exhibited new-onset positivity for LA. Therefore, the preconception management of patients with SLE should not only ensure sustained disease stability for at least 6 months and necessary adjustments to immunosuppressive agents, but also involve stratification of therapeutic strategies based on their history of adverse pregnancy events, thrombotic events, and the aPL profile. aPLs-seropositive patients should receive long-term low-dose aspirin therapy, with the initiation of low-molecular-weight heparin therapy during controlled ovarian stimulation (40, 41). Hydroxychloroquine, a cornerstone medication in the treatment of SLE, not only exhibits immunomodulatory effects but also reduces the risk of thrombotic events (42). This protective effect is exemplified by Patient 5, who had persistent aPLs seropositivity but remained thrombosis-free while on hydroxychloroquine and low-dose aspirin. Conversely, Patient 7 developed severe venous thrombosis during glucocorticoid monotherapy. Notably, Patient 4, who received no treatment for the primary disease, experienced three failed ART attempts and two severe SLE flares.

In summary, patients with SLE undergoing ovarian stimulation should be managed in strict compliance with established guidelines through close collaboration between reproductive medicine and rheumatology. This collaboration requires a thorough consideration of the adverse effects of estrogen level changes on SLE and the constant priority of maternal safety.

## Data Availability

The original contributions presented in the study are included in the article/supplementary material. Further inquiries can be directed to the corresponding authors.
